# La problématique du coût des nouvelles thérapeutiques en oncologie: qu'en-est-il du Maroc?

**DOI:** 10.11604/pamj.2016.24.51.9512

**Published:** 2016-05-11

**Authors:** Sami Aziz Brahmi, Ziani Fatima Zahra, Youssef Seddik, Said Afqir

**Affiliations:** 1 Service d'Oncologie Médicale, Centre Hospitalier Mohammed VI, Oujda, Maroc; 2 Service d'Oncologie Médicale, Centre Hospitalier Hassan II, Fès, Maroc

**Keywords:** Cancer, new therapies, cost, public health, Cancer, new therapies, cost, public health

## Abstract

Le cancer est un problème majeur de santé public en Afrique. Les progrès réalisé dans le traitement des cancers ces dix dernières années est indéniable. L’émergence des thérapies ciblés en oncologie a permit de modifier l'histoire naturelle de certains cancers réputés de mauvais pronostic. En dépit de leurs efficacité, ces thérapeutiques pose un problème majeur de coût qui les rend inaccessible à la majorité des patients dans les pays en voie développement. Au Maroc, le cancer est reconnu comme a une affection de longue durée et les patients bénéficient de ce fait d'une couverture médicale totale. L'implication de la société civile a permis aussi d'améliorer la prise en charge ainsi qu'un accès plus élargi aux médicaments innovants pour les patients les plus démunis.

## Abstract (en)

Cancer is a major public health problem in Africa. The progress in cancer treatment over the past decade is undeniable. The emergence of targeted therapies in oncology made it possible to modify the natural history of some cancers associated with a poor prognosis. Despite their efficiency, these therapies pose a major health care problem that makes them inaccessible to most patients in developing countries. In Morocco, cancer is considered a long-term condition and for this reasons patients have health insurance coverage. The involvement of civil society made it possible even to improve therapy management as well as a broader poor patient's access to innovative medicines.

## Aux editeurs du Journal Panafricain de Médecine

Le cancer est devenu un problème de santé public majeur en Afrique. En 2012 on a estimé le nombre de nouveaux cas de cancer à 847 000 (soit un taux de 6.0% à l’échelle mondiale) et 591000 de décès par cancer (soit un taux de 7.2% à l’échelle mondiale) [1]. Le nombre de nouveaux cas augmentera de 70% entre 2012 et 2020, une croissance plus rapide que n'importe quelle région au Monde [2]. Plusieurs facteurs ont contribués à l'augmentation de l'incidence des cas de cancer en Afrique notamment le vieillissement de la population et l'apparition de nouveaux facteurs de risque du à la transition économique que connaissent certains pays d'Afrique. Néanmoins, les ressources disponibles pour combattre ce fléau manquent en Afrique à cause du faible niveau économique et l'intérêt porté par les gouvernement à d'autres problèmes de santé public comme les maladies infectieuses jugés plus graves. Les progrès réalisé dans le traitement des cancers ces dix dernières années est indéniable. L’émergence des thérapies ciblés en oncologie a permit de modifiés l'histoire naturelles de certains cancers réputés de mauvais pronostic. En situation métastasique on assisté de plus en plus à une chronicisation de la maladie grâce a ces nouvelles thérapeutiques. Pour le cancer du sein en situation métastatique surexprimant HER2 (Human epithelial receptor 2), on dispose de deux thérapies ciblée qui sont le trastuzumab et le pertuzumab qui a permit de prolonger le survie globale de 15.7 mois en association avec la chimiothérapie [3].

En dépit de leurs efficacité, ces thérapeutiques pose un problème majeur de coût qui les rend inaccessible à la majorité des patients dans les pays en voie développement. Dans étude récente, de coût efficacité cette problématique semble présente même dans les pays développés comme les états unis, en effet selon cette étude l'association trastuzumab-pertuzumab dans le cancer du sein métastatique le rapport coût efficacité n'est pas favorable à ces thérapeutiques [4]. A l'instar de la plupart des pays africains, le cancer constitue un problème de santé public au Maroc, environ 30000 nouveaux cas sont diagnostiqués chaque année [5]. Le cancer est reconnu au Maroc comme une affection de longue durée, bénéficiant ainsi d'une couverture totale. Le régime marocain de protection sociale couvre les salariés du secteur public et ceux du secteur privé. Il s'agit du système d'assurance Maladie Obligatoire (AMO) qui a été est institué en 2002 par la loi 65.00 portant code de la couverture médicale de base instituant la couverture médicale obligatoire de base garantissant l'accès universel aux soins de santé. Un deuxième système; le régime d'aide médicale (RAMED) couvre les sujets les plus démunis. Environ deux tiers de la population est couvert. Les médicaments sont commercialisés après obtention d'une autorisation de mise sur le marché (AMM). Plusieurs thérapies ciblées onéreuses ont obtenues l'AMM au Maroc notamment: le bevacizumab, le trastuzumab, l'imatinib, l'erlotinib et le sunitinib.

Par ailleurs, depuis sa création en 2005, la Fondation Lalla Salma de lutte contre le cancer œuvre a fait de la lutte contre le cancer une priorité de santé publique au Maroc et dans la région. Cette fondation a constitué un levier pour une meilleure prise en charge des patients atteints de cancer à travers la promulgation d'un plan Cancer. Plusieurs actions ont été visées notamment: un meilleurs accès aux médicaments innovants pour les plus démunis, la création de centres d'oncologie et de pôles d'excellence, la promotion de la recherche ainsi que les campagnes de dépistage et de prévention. Pour permettre aux patients démunis de bénéficier des médicaments de dernière génération, un protocole d'accord a été signé entre la Fondation Lalla Salma et les laboratoires pharmaceutiques permettant d'offrir, à travers les différents centres d'oncologie publics, des médicaments les plus innovants en cancérologie.

## Conclusion

Les médicaments innovant en oncologie constituent un espoir d'une meilleur survie et qualité de vie pour les patients. En Afrique, l'inaccessibilité à ces traitements est une perte de chance pour des millions de patients. L'implication des gouvernements ainsi que de la société civile est primordiale pour garantir une prise en charge optimale.
